# Perampanel for Early Seizure Prophylaxis After Severe Traumatic Brain Injury: A Retrospective Comparative Study with Levetiracetam

**DOI:** 10.1177/2689288X251372910

**Published:** 2025-09-03

**Authors:** Hidetaka Onda, Takuya Nishino, Shoji Yokobori

**Affiliations:** ^1^ Department of Disaster and Emergency Medicine, Kochi University, Nankoku, Japan.; ^2^ Department of Emergency and Critical Care Center, Nippon Medical School Hospital, Tokyo, Japan.; ^3^ Department of Health Policy and Management, Nippon Medical School, Tokyo, Japan.

**Keywords:** post-traumatic epilepsy, prophylactic therapy, intensive care, neuroprotection

## Abstract

Early post-traumatic seizures (EPTS) after severe traumatic brain injury (TBI) are linked to poor neurological outcomes. While levetiracetam (LEV) is commonly used for seizure prophylaxis, perampanel (PER), an AMPA receptor antagonist, is gaining interest due to its potential neuroprotective effects. This retrospective cohort study included adults (≥18 years) admitted to a tertiary trauma center between 2018 and 2025 who received LEV or PER within 12 h post-injury (*N* = 200; LEV, *n* = 145; PER, *n* = 55). Monotherapy was initiated with either drug. The primary outcome was EPTS incidence within 7 days. Secondary outcomes included mechanical ventilation duration, hospital stay, and Glasgow Outcome Scale (GOS) score. Analyses included Firth’s logistic regression and inverse probability of treatment weighting (IPTW) adjusted for key clinical confounders. EPTS occurred at 15.9% (LEV) versus 1.8% (PER). PER significantly reduced seizure risk (Firth-adjusted OR = 0.158; 95% CI, 0.017–0.644; *p* = 0.007), which was confirmed by IPTW (OR = 0.112; 95% CI, 0.014–0.873; *p* = 0.037). PER also shortened ventilation duration (IRR = 0.515; 95% CI, 0.303–0.907; *p* = 0.015). GOS and hospital stay did not differ significantly. No adverse events occurred in either group. PER significantly reduced EPTS and mechanical ventilation duration compared with LEV. The safety and utility of PER in acute care suggest it might be a valuable alternative for seizure prophylaxis in patients with severe TBI.

## Introduction

The prevention and management of acute symptomatic seizures following severe traumatic brain injury (TBI) are critical components of acute neurocritical care, given their potential impact on neurological outcomes.^1^ Current international and national guidelines, including those from the Brain Trauma Foundation and the Japan Society of Neurotraumatology, recommend early prophylactic administration of antiepileptic drugs such as phenytoin or levetiracetam (LEV) during the acute post-injury period, and this practice has been widely adopted in clinical settings.^2–4^

Acute post-traumatic seizures are thought to result from various pathophysiological changes, including increased cerebral metabolic demand, elevated intracranial pressure, disrupted cerebral perfusion, blood–brain barrier breakdown, and enhanced neuroinflammatory responses.^5–9^ These alterations, beyond the initial structural damage, may lead to the progression of secondary brain injury through mechanisms such as spreading depolarization, thereby impairing functional recovery.^10,11^

These seizures are associated with a worse neurological prognosis, prolonged intensive care unit (ICU) stays, and increased mortality.^12^ Moreover, early seizures are a known risk factor for the development of late post-traumatic epilepsy, underscoring the importance of early seizure control.^13,14^ Reported rates of early seizures vary widely, from 5% to 25%, depending on factors such as injury severity, lesion type, age, and comorbidities.^15,16^ In patients with severe TBI, the incidence is notably higher, justifying the need for more aggressive prophylactic intervention.^17^

Phenytoin has traditionally been used for early seizure prevention; however, concerns regarding adverse effects and the need for serum level monitoring have led to the increasing use of LEV, which is not without limitations, as variability in seizure control and neuropsychiatric adverse effects have been reported. These issues highlight the need for alternative agents with improved efficacy and safety.^18–20^

Perampanel (PER), a selective noncompetitive α-amino-3-hydroxy-5-methyl-4-isoxazolepropionic acid (AMPA) receptor antagonist, has recently garnered interest for its potential neuroprotective effects.^21^ Preclinical studies have demonstrated its ability to attenuate cerebral edema, reduce contusion volume, and suppress neuronal cell death in models of acute TBI.^22^ Furthermore, its long half-life and once-daily dosing offer practical advantages for acute care settings.

Despite these promising pharmacological properties, the clinical efficacy of PER in the acute management of severe TBI remains largely unestablished. In particular, its effectiveness for preventing early seizures, as well as its safety and impact on neurological outcomes, has yet to be systematically evaluated in clinical settings.

## Objective

This retrospective study aimed to evaluate the effectiveness of PER monotherapy for early seizure prophylaxis in patients with severe TBI who were admitted to our institution between May 2024 and April 2025. Outcomes were compared with those of a historical cohort who received LEV for the same indications between 2018 and 2024. We assessed the incidence of early post-traumatic seizures (EPTS) as the primary outcome and examined clinical characteristics between the PER- and LEV-treated cohorts, including vital signs on admission, initial laboratory findings, computed tomography (CT) imaging features, and injury severity scores (Abbreviated Injury Scale [AIS] and Injury Severity Score [ISS]).

Through this comparative analysis, we sought to clarify the clinical utility of PER in the acute phase of TBI and determine its potential as an alternative strategy to LEV for seizure prophylaxis, with the goal of contributing to improved therapeutic decision-making and outcomes in neurocritical care.

## Methods

### Patients

This retrospective study included patients with TBI who were transported to our tertiary emergency medical center in Tokyo between 2018 and 2025. Only patients classified as severely injured according to Tokyo Metropolitan Emergency Transport criteria were admitted. Exclusion criteria included age <18 years, cardiopulmonary arrest on arrival, a history of antiseizure medication use, pregnancy, and alcohol dependence. Eligible cases were limited to those who received prophylactic antiseizure medication for EPTS prevention.

Antiseizure drug administration was determined by treatment era: LEV was administered between 2018 and 2024, and PER was used from 2024 to 2025. All patients received intravenous formulations of either LEV or PER, both of which are approved and reimbursed under the Japanese national health insurance system. From 2018 to 2024, LEV was administered intravenously at a dose of 500 mg twice daily (total daily dose: 1000 mg). Intravenous PER, introduced into clinical practice in 2024 following national approval, was administered intravenously at a dose of 2 mg once daily. Drug administration began within 12 h post-injury in all cases, and dosing was adjusted at the discretion of neurocritical care physicians. Upon admission, all patients underwent evaluation of vital signs, laboratory testing, and head CT. Multidisciplinary management in the ICU was provided by the neurocritical care team. Continuous electroencephalographic (cEEG) monitoring was initiated in patients with suspected seizure activity at the discretion of the attending intensivist. During the study period, there were no changes in the institutional protocols for the acute management and neurocritical care of patients with TBI, except for the antiseizure medication strategy. The same board-certified neurointensivists consistently provided care throughout the study period.

### Data Collection

On arrival, age, sex, systolic blood pressure, heart rate, and level of consciousness (Glasgow Coma Scale [GCS]) were recorded. Laboratory results and head CT findings were reviewed for the presence, number, and size of cerebral contusions; presence of subarachnoid hemorrhage, subdural hematoma, interhemispheric subdural hematoma, bilateral subdural hematoma, and epidural hematoma; skull fractures; and midline shift ≥5 mm.

### Definition of Early Seizures

Early seizures were defined as (1) a documented clinical seizure noted by a physician or nurse followed by administration of additional antiseizure therapy, including benzodiazepines; or (2) seizure activity diagnosed by neurointensivists based on cEEG findings in patients with clinical suspicion of nonconvulsive status epilepticus.

### Outcomes

The primary outcome was the incidence of EPTS within 7 days of injury. Secondary outcomes included length of hospital stay, Glasgow Outcome Scale (GOS) at discharge, and duration of mechanical ventilation. These outcomes were compared between the LEV and PER groups.

### Statistical Analysis

Continuous variables were summarized as medians with interquartile ranges, and categorical variables as counts with percentages. Comparisons between groups were performed using Fisher’s exact test or the chi-square test for categorical variables and the Wilcoxon rank-sum or *t-*test for continuous variables, as appropriate. To assess covariate balance, standardized mean differences (SMDs) were calculated, with |SMD| < 0.10 indicating acceptable balance.

Unadjusted seizure event rates were analyzed using Fisher’s exact test. To account for sparse events and small sample bias, Firth’s penalized logistic regression was applied with PER as the main explanatory variable and age, sex, AIS, ISS, and GCS as covariates, yielding odds ratios (ORs) with 95% confidence intervals (CIs). In addition, inverse probability of treatment weighting (IPTW) was conducted using the same covariates to estimate the average treatment effect.

For secondary outcomes (ventilation days and length of hospital stay), negative binomial regression was used due to overdispersion. Incidence rate ratios (IRRs) with 95% CIs were reported.

All analyses were performed using StatFlex Plus (Version X.X; ViewFlex Co., Tokyo, Japan), with 2-sided significance set at *p* < 0.05.

### Ethical Considerations

This study protocol was approved by the Institutional Review Board of Nippon Medical School Hospital (approval no. O2022612) and conducted in accordance with the 1975 Declaration of Helsinki and its later amendments. Informed consent was obtained using an opt-out method at the time of admission, allowing for the use of anonymized data. The requirement for additional informed consent for this retrospective analysis was waived by the ethics committee.

## Results

A total of 200 patients with blunt TBI who were transported to our center during the study period were included in the analysis. Patients with a history of epilepsy, chronic use of antiseizure medications, pregnancy, or alcohol dependence were excluded. Among the included cases, 145 received LEV, and 55 received PER. There were no significant differences in baseline characteristics between the two groups. All patients underwent head CT on admission, and findings such as cerebral contusions, acute subdural or epidural hematomas, traumatic subarachnoid hemorrhage, skull fractures, and midline shift were assessed. No significant intergroup differences in radiological findings were observed (Table 1).

**Table 1.** Comparison of Characteristics Between the LEV and PER Groups

Parameter	LEV	PER	*p* value
Cases, *n*	145	55	
Early seizure	23	1	0.012
Age, years	61.5 (23.2)	62.4 (20.8)	0.919
Male, *n*	93 (76%)	21 (84%)	0.766
GCS score	10 (6–13)	8 (4–13.8)	0.616
Operation, *n*	62 (32%)	24 (44%)	0.457
Favorable outcome, *n*	81 (56%)	35 (64%)	0.612
Death, *n*	14 (9%)	5 (9%)	0.912
AIS (head)	4 (3–5)	4 (3–5)	0.956
ISS	18 (16–25)	21 (14.5–26.0)	0.451
Length of hospital stay, days	19.0 (11–32.3)	17.5 (10.0–25.0)	0.181
Dependent on ventilation	10.0 (3–20)	3.0 (1–8)	0.015
SAH, *n*	111 (77%)	39 (71%)	0.754
SDH, *n*	105 (72%)	38 (75%)	0.849
EDH, *n*	25 (17%)	9 (16%)	0.901
Contusion, *n*	75 (52%)	26 (47%)	0.745
Skull fracture, *n*	34 (23%)	12 (22%)	0.846
Midline shift, n	45 (31%)	20 (36%)	0.611

AIS, Abbreviated Injury Scale; EDH, epidural hematoma; GCS, Glasgow Coma Scale; ISS, Injury Severity Score; LEV, levetiracetam; PER, perampanel; SAH, subarachnoid hemorrhage; SDH, subdural hematoma.

Baseline demographics and injury severity were well balanced between the groups (all LEV vs. PER): (mean ± SD: age, 61.2 ± 23.2 vs. 62.4 ± 20.8 years), head AIS (4.01 ± 0.78 vs. 4.00 ± 0.79), ISS (21.01 ± 9.27 vs. 20.49 ± 7.14), GCS (9.65 ± 3.92 vs. 9.18 ± 4.65), and male sex (77.9% vs. 72.7%). SMDs were all <0.10 except for sex (SMD = 0.12), indicating no clinically meaningful imbalance (Table 2).

**Table 2.** Standardized Mean Differences Between the LEV and PER Groups

Parameter	Overall	LEV	PER	SMD
Cases, *n*	200	145	55	
Age, years	61.76 (22.51)	61.51 (23.18)	62.40 (20.81)	0.040
Male, *n* (%)	153 (76.5)	113 (77.9)	40 (72.7)	0.121
AIS (head)	4.00 (0.78)	4.01 (0.78)	4.00 (0.79)	0.009
ISS	20.87 (8.72)	21.01 (9.27)	20.49 (7.14)	0.063
GCS score	9.52 (4.13)	9.65 (3.92)	9.18 (4.65)	0.108
Dependent on ventilation	6.84 (9.79)	8.19 (10.58)	3.27 (6.08)	0.570
Length of hospital stay, days	21.66 (13.70)	22.64 (14.37)	19.02 (11.43)	0.279

AIS, Abbreviated Injury Scale; GCS, Glasgow Coma Scale; ISS, Injury Severity Score; LEV, levetiracetam; PER, perampanel; SMD, standardized mean difference.

Early seizures within 7 days of injury occurred in 23 patients (15.9%) in the LEV group and 1 patient (1.8%) in the PER group. Unadjusted analysis demonstrated a significant reduction in seizure incidence in the PER group (OR = 0.110; 95% CI, 0.003–0.720; *p* = 0.010). After adjusting for covariates (age, sex, AIS, ISS, and GCS) using Firth logistic regression, PER use remained significantly associated with reduced seizure risk (adjusted OR = 0.158; 95% CI, 0.017–0.644; *p* = 0.007). Consistent findings were obtained in the IPTW-adjusted analysis (OR = 0.112; 95% CI, 0.014–0.873; *p* = 0.037) (Table 3).

**Table 3.** Evaluation of the Effectiveness of PER (Primary Analysis)

Model	Odds ratio	95% CI	*p* value
Unadjusted analysis (Fisher)	0.110	0.003–0.720	0.010
Firth	0.158	0.017–0.644	0.007
IPTW	0.112	0.014–0.873	0.037

Regarding seizure-related findings, cEEG monitoring was performed in 109 patients (75.2%) in the LEV group and 38 patients (84.4%) in the PER group. These cases were primarily postoperative patients and patients with a Glasgow Coma Scale (GCS) score of ≤12. The median duration of cEEG monitoring was 3 days. Clinical seizures were observed in 12 patients in the LEV group and 1 patient in the PER group; electrographic seizures were detected in 11 patients in the LEV group and 0 in the PER group (Table 4).

**Table 4.** Seizure-related Findings and cEEG Monitoring Characteristics in the LEV and PER Groups

Parameter	Overall	LEV	PER	*p* value
Cases, *n*	200	145	55	
Early seizure	24	23	1	0.012
Patients with cEEG	147	109	38	0.490
Median cEEG duration (Day)	3 (2–5)	3 (2–5)	4	1
Clinical seizures	13	12	1	1
Electrographic seizures	11	11	0	1

cEEG, continuous electroencephalographic monitoring; LEV, levetiracetam; PER, perampanel.

**FIG. 1.**
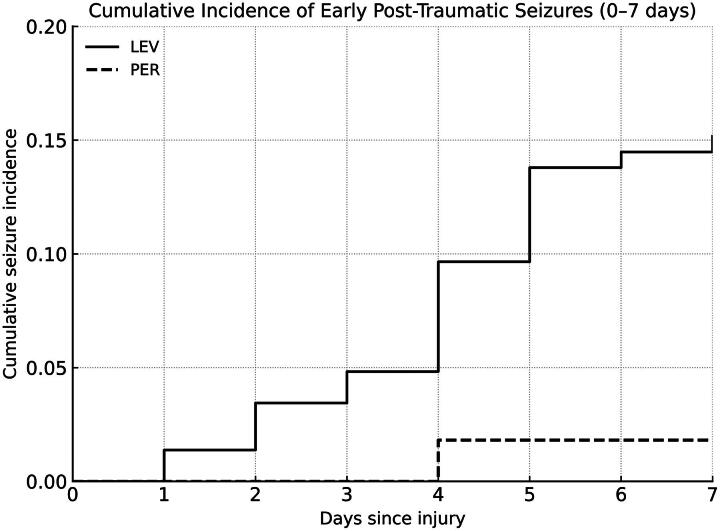
Cumulative incidence of early post-traumatic seizures (EPTS) within 7 days after injury. This figure shows the cumulative incidence of seizures in patients with severe traumatic brain injury who received levetiracetam (LEV) or perampanel (PER) for prophylactic treatment. In the LEV group, seizures occurred progressively, reaching a cumulative incidence of 15.9% by day 7. In contrast, only one seizure (1.8%) was observed in the PER group, on day 3. Patients who remained seizure-free were censored on day 7. Throughout the observation period, the PER group demonstrated a significantly lower cumulative incidence of EPTS.

The incidence of EPTS within 7 days after injury was evaluated in both groups (Fig. 1). In the LEV group, seizures occurred in a gradually increasing manner, with a cumulative incidence reaching 15.9% (23/145) by day 7. In contrast, only 1 seizure (1.8%) was observed in the PER group, on day 3, and no additional seizures were identified during the remainder of the observation period. Patients who remained seizure-free through day 7 were treated as censored, and the seizure incidence was estimated over time, using a time-to-event approach. Throughout the observation period, the PER group exhibited a significantly lower cumulative seizure incidence compared with the LEV group. Regarding secondary outcomes, PER administration was associated with a significantly shorter duration of mechanical ventilation (IRR = 0.515; 95% CI, 0.303–0.907; *p* = 0.015) in negative binomial regression analysis. Length of hospital stay showed a tendency toward reduction in the PER group but did not reach statistical significance (IRR = 0.845; 95% CI, 0.699–1.026; *p* = 0.085). Favorable neurological outcomes at discharge (GOS 4–5) were observed in 56% of patients in the LEV group and 64% in the PER group; however, this difference was not statistically significant (*p* = 0.612) (Table 5).

**Table 5.** Analysis of Secondary Outcomes of the PER Group

Outcome	Estimate	Conf. low	Conf. high	*p* value
Dependent on ventilation	0.515	0.303	0.907	0.015
Length of hospital stay, days	0.845	0.699	10.026	0.085

PER, perampanel.

Taken together, PER administration was associated with a significantly lower incidence of EPTS and reduced duration of ventilation in comparison with LEV. These results remained robust after multiple adjustments for confounders, providing supportive evidence for the efficacy of PER in this setting.

### ICU Management

All patients were admitted to the ICU and received standardized care according to contemporary neurocritical care guidelines. Emergency surgery was performed as needed on admission or within 7 days of presentation. All patients received initial antiseizure medication within 12 h post-injury. Intravenous formulations were used initially, followed by oral administration when feasible. Serum drug levels were not routinely measured. No adverse events, including rash, hematological abnormalities, or hepatotoxicity, were observed in either group.

This study demonstrated that PER monotherapy was effective in reducing early seizure incidence in patients with severe TBI, with no reported adverse events. Compared with the historical LEV cohort, PER appeared to offer favorable safety and efficacy profiles in the acute neurocritical setting.

## Discussion

PER is a selective, noncompetitive antagonist of the AMPA receptor and has the unique ability to directly inhibit glutamate-dependent excitatory signaling, which is markedly upregulated following TBI.^22,23^ In contrast to conventional antiseizure medications, which primarily target synaptic transmission or voltage-gated ion channels, PER blocks the excitotoxic cascade at the receptor level, offering a mechanistically upstream approach to neuroprotection.^24^

In animal models, PER has been shown to attenuate cerebral edema, reduce neuronal cell death, and preserve cerebral blood flow, suggesting that it may improve neurological outcomes beyond seizure suppression alone.^25,26^ These findings highlight its potential as a neuroprotective agent in acute TBI management.

Although LEV has been widely used as a standard prophylactic antiseizure medication in TBI due to its favorable safety profile and ease of administration, its neuroprotective effects appear to be limited, and neuropsychiatric adverse effects have been reported in some cases.^15,27,28^ Notably, no adverse events were observed in the PER group in the present study, further supporting its safety in the acute phase of severe TBI.

The selection of antiseizure medications should consider not only efficacy in seizure prevention but also neuroprotective properties, safety, and practical applicability. While phenytoin was long considered the standard of care, the need for therapeutic drug monitoring, along with its cardiotoxicity and risk of severe dermatologic reactions such as Stevens–Johnson syndrome, makes it less suitable for critically ill patients. LEV has largely replaced phenytoin due to its simplicity and favorable tolerability, with randomized controlled trials showing comparable efficacy for early seizure prevention and fewer adverse effects.^29–33^ However, LEV may offer limited protection against secondary brain injury.

PER, through its AMPA receptor antagonism, can directly modulate excitotoxicity after TBI, offering additional benefits such as reduction of cerebral edema, protection of the blood–brain barrier, and inhibition of neuronal death.^34^ Preclinical studies have suggested that PER may also prevent the progression of secondary brain injury.^35,36^ Furthermore, the availability of intravenous formulations and the feasibility of once-daily dosing present considerable advantages in critical care environments, allowing for stable drug delivery with minimal intervention.^37^

Thus, when evaluating pharmacologic strategies for EPTS prevention, consideration should extend beyond seizure suppression to include neuroprotective capacity and logistical feasibility. The observed trend toward lower seizure incidence and shorter ventilation duration in the PER group provides initial clinical evidence supporting its utility in this setting.

The shortened duration of mechanical ventilation in the PER group is likely attributable to the lower incidence of early seizures observed in this group. In addition, our protocol mandated the administration of antiseizure medications within 12 h of injury, a factor that may have contributed to improved respiratory outcomes. Previous studies have also suggested that the timing of administration of antiseizure medications and the specific agent used may influence ventilation requirements, although causality remains to be clarified.^38–40^

Future studies should incorporate neurophysiological monitoring, such as cEEG and brain tissue oxygenation measurement, to more objectively assess the neuroprotective effects of PER.^41,42^ Moreover, subgroup analyses stratified by injury severity and mechanism of trauma will be important in identifying the patient populations most likely to benefit from this intervention.^43,44^

### Limitations

This study has several limitations. First, the retrospective, single-center design may limit generalizability, as institutional practices and regional case characteristics could affect treatment and outcomes. Second, the sample size was modest, limiting the statistical power to detect small effect sizes. Third, as a tertiary trauma center, our facility receives disproportionately severe cases, which may introduce bias in estimating associations between TBI and functional outcomes. Multicenter, prospective randomized controlled trials are warranted to validate our findings.

## Conclusions

In this cohort, PER monotherapy was associated with a markedly lower incidence of EPTS and a significantly shorter duration of mechanical ventilation in comparison to LEV. Baseline characteristics were well balanced between groups, and results remained consistent after multiple statistical adjustments. These findings support the efficacy of PER and suggest its potential as a promising alternative for seizure prophylaxis in patients with severe TBI.

## Data Availability

The data supporting the findings of this study are not publicly available because of privacy and ethical restrictions but can be obtained from the corresponding author upon reasonable request.

## Abbreviations Used

AIS: Abbreviated Injury Scale
AMPA: α-amino-3-hydroxy-5-methyl-4-isoxazolepropionic acid
cEEG: continuous electroencephalographic
CI: confidence interval
CT: computed tomography
EDH: epidural hematoma
EPTS: Early post-traumatic seizures
GCS: Glasgow Coma Scale
GOS: Glasgow Outcome Scale
IPTW: inverse probability of treatment weighting
ICU: intensive care unit
IRR: Incidence rate ratio
ISS: Injury Severity Score
LEV: levetiracetam
OR: odds ratio
PER: perampanel
SAH: subarachnoid hemorrhage
SDH: subdural hematoma
SMD: standardized mean differences
TBI: severe traumatic brain injury
